# Pathological study of the tumor microenvironment after neoadjuvant therapy in hepatocellular carcinoma: Difference of TACE combined with antiangiogenics and immunotherapy

**DOI:** 10.1097/HC9.0000000000000787

**Published:** 2025-08-29

**Authors:** Xintao He, Yanhong Liu, Tianyi Dai, Aihua Yang, Jianan Shen, Zexuan Hui, Jie Shen, Jun Chen

**Affiliations:** 1 Department of Pathology, Nanjing Drum Tower Hospital Clinical College of Nanjing University of Chinese Medicine, Nanjing, China; 2 Department of Biobank, Nanjing Drum Tower Hospital Clinical College of Nanjing University of Chinese Medicine, Nanjing, China; 3 Department of Oncology, Nanjing Drum Tower Hospital Clinical College of Nanjing University of Chinese Medicine, Nanjing, China

**Keywords:** HCC, neoadjuvant therapy, vessels that encapsulate tumor clusters, transcatheter arterial chemoembolization, tumor microenvironment

## Abstract

**Background:**

HCC is the leading form of primary liver cancer worldwide. Transcatheter arterial chemoembolization (T) is commonly used to treat unresectable tumors. T combined with antiangiogenic therapy and immunotherapy (AI) has shown significant progress in neoadjuvant treatment, although the underlying mechanisms remain unclear. This study aimed to explore the reasons for the enhanced efficacy of T+AI from a pathological perspective in the context of HCC.

**Methods:**

A retrospective analysis was conducted on 49 patients with HCC who were treated with T before surgical resection. Twenty-three patients received T+AI, while 26 received only T. Immunohistochemistry was performed to evaluate clinical data, including disease-free survival. Immune cells were recorded based on 4 methods, including tumor-infiltrating lymphocyte (TIL) percentage (the percentage of positive lymphocytes in the central area of the tumor) and the other 3 methods. Blood vessels were classified on the basis of the presence of VETC (vessels that encapsulate tumor clusters).

**Results:**

The group analysis results suggested that disease-free survival in the T+AI group was significantly better than that in the T group. Analysis revealed that CD8+TILs were a prognostic factor for neoadjuvant treatment and lower carbonic anhydrase 9 and VETC positivity in the T+AI group, with significant differences in immune cell infiltration and vascular classification. VETC positivity was associated with higher residual tumor rates and lower CD8+TIL levels.

**Conclusions:**

Pathological assessment of CD8+TILs in cancer tissues may serve as an important indicator for evaluating the efficacy of neoadjuvant therapy in HCC. The presence of VETC and carbonic anhydrase 9 may also affect the efficacy of neoadjuvant therapy and could potentially serve as indicators.

## Graphical abstract

**Figure:**
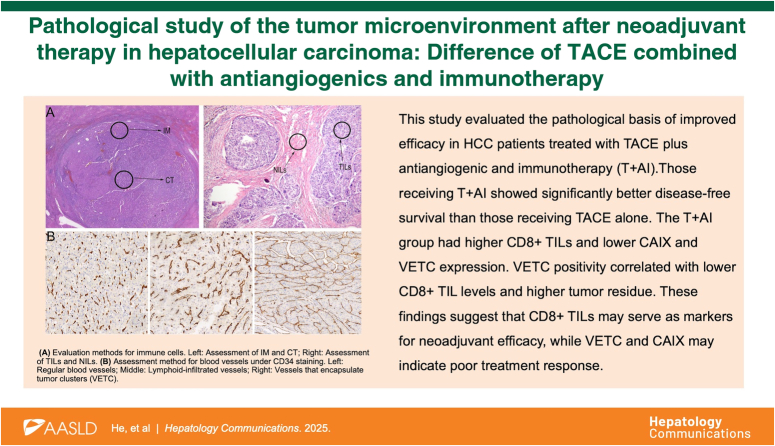

## INTRODUCTION

HCC is a highly lethal tumor with an abundant blood supply.^1^ China has a high incidence of liver cancer, accounting for ~50% of the world’s new liver cancer cases annually.^2^ Surgery is the first-line treatment recommended by the NCCN guidelines^3^; however, only 30% of patients are eligible for resection at the time of diagnosis, and the overall prognosis for unresectable HCC is poor. For patients who are not surgical candidates, treatment options have evolved from sorafenib-targeted and antiangiogenesis-targeted therapies to immunotherapy and combination therapies,^4^ continually improving the therapeutic landscape for liver cancer.

In addition to these treatments, neoadjuvant therapy—using various approaches to reduce tumor burden before surgery, increase the chance of resection, and reduce postoperative recurrence—has become an important option for HCC treatment.^5^

Owing to the rich blood supply of HCC, transarterial chemoembolization (TACE) is widely used as a neoadjuvant treatment. However, TACE alone has shown limited efficacy. In a study of 832 patients with initially unresectable liver cancer, the conversion rate was only 9.9%.^6^ In contrast, a combination of TACE with antiangiogenesis drugs and immunotherapy significantly outperformed TACE alone, with a conversion rate of 52.6% in a prospective study.^7^

The evaluation of neoadjuvant therapy in HCC primarily focuses on tumor characteristics and the extent of residual disease. The pathologic complete response (PCR) and major pathologic response (MPR) are key indicators used to assess the efficacy of neoadjuvant treatment.^8^ While PCR is well defined, the definition of MPR remains controversial. Allard et al suggested that a residual cancer tissue <10% indicates a good prognosis for patients who undergo resection or liver transplantation after neoadjuvant therapy. However, other studies have proposed that defining MPR as <30% residual cancer may be a better predictor of favorable outcomes. The study of the tumor microenvironment is also gaining traction, with markers such as immunoscores in colorectal cancer^9^ and sTILs (Stromal Tumor-Infiltrating xLymphocytes) in breast cancer, which are valuable in clinical practice.

In terms of assessing the tumor microenvironment in HCC, markers such as TMB, MSI-H, and PD-L1^10^ have a limited predictive value for drug efficacy, recurrence, and long-term survival. However, emerging pathological features such as tertiary lymphoid structures^11^ and vessels that encapsulate tumor clusters (VETC)^12^ in HCC have shown promising prognostic significance.

Thus, we aimed to investigate cases at our hospital that have undergone neoadjuvant therapy, focusing on identifying pathological factors that influence post-treatment prognosis and understanding why the combination of TACE and immunotherapy may yield better outcomes.

## METHODS

### Patient material

A retrospective analysis of 76 patients who were treated with TACE before resection and had a confirmed histological diagnosis of HCC after radical operation at Nanjing Drum Tower Hospital, undergoing interventional therapy from January 2019 to December 2022, was conducted. Twenty-seven patients were excluded owing to loss of follow-up, and 49 patients were enrolled in the study. Twenty-three patients underwent immunotherapy before surgery, 23 cases were treated with TACE (transcatheter arterial chemoembolization) combined with immunotherapy (all of whom used PD1/PDL1 inhibitor), and 26 patients were treated with TACE. Cases were divided into 2 groups (T, T+AI [antiangiogenic and immunotherapy]) according to whether immunotherapy and antiangiogenic agents were added, and were retrospectively studied. This study was approved by Nanjing Drumtower Hospital Ethics Committee (No:2013-081-12).

### Clinical and prognostic data

Clinical information, including laboratory blood tests, Barcelona Clinic Liver Cancer (BCLC) stage, overall survival (OS), and disease-free survival (DFS), was meticulously collected from the patients.

**FIGURE 1:**
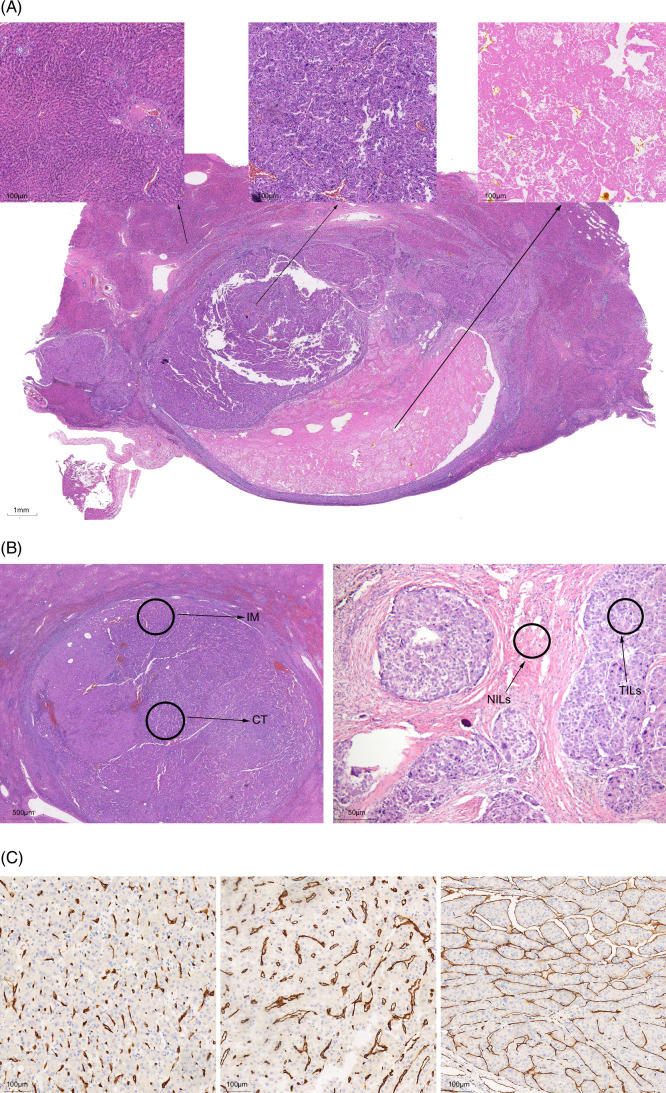
Assessment methods for tumor sections. Evaluation method for tumor residue rate. (A) left: normal liver tissue; middle: residual cancer area; right: necrotic area. (B) Evaluation methods for immune cells. Left: Assessment of IM and CT; Right: Assessment of TILs and nontumorous tissue-infiltrating lymphocytes. (C) Assessment method for blood vessels under CD34 staining. Left: Regular blood vessels; middle: lymphoid-infiltrated vessels; right: vessels that encapsulate tumor clusters (VETC).

Pathological evaluation was performed by a pathologist on all hematoxylin and eosin (HE) slides. Following treatment, the tumor displayed distinct histological features: (1) residual cancer tissue, (2) areas of necrosis, and (3) normal tissue (Figure 1A). A pathological complete response (PCR) was considered when the residual tumor tissue reached 0%, and residual tumor tissue of at least 30% was considered a major pathological response (MPR).

Tumor stages were evaluated using the tumor-node-metastasis staging system (TNM, 8th edition), and HCC cell differentiation was assessed using the Edmondson-Steiner Method. Pathological assessments were conducted in accordance with World Health Organization (WHO) classification by pathologists who were blinded to the patients’ clinical outcomes.

### Immunohistochemistry

Immunohistochemical staining of tumor tissues from patients was performed using antibodies targeting tumor markers (Ki67, PD-L1, CK19, and CA9), immune cell markers (CD4, CD8, CD20, PD1, FoxP3, and CD66b), and a vessel marker (CD34). The marker proteins were visualized using diaminobenzidine, and the nuclei were counterstained with hematoxylin. Tumor specimens were evaluated using hematoxylin and eosin (H&E) staining.

CK19 and CA9 were classified as positive or negative, Ki67 was evaluated based on the proportion of tumor cells expressing Ki67, and PD-L1 was assessed using the Tumor Proportion Score and Combined Positive Score.

For immune cell markers, evaluation included 4 methods:

1. Positive cells in the invasion margin (IM): positive cells located in the outer layer of the tumor.
2. Positive cells in the center of the tumor (CT): positive cells in the central region of the tumor are not in direct contact with stroma or tumor cells.
3. Tumor-infiltrating lymphocytes (TILs): Positive cells that directly infiltrate tumor cells.
4. Nontumorous tissue-infiltrating lymphocytes (NILs): Positive cells in the nontumorous stroma within the tumor center.

IM and CT were evaluated by the average marker expression in three high-power fields, whereas TILs and NILs were counted in low-power fields. The cases reached PCR will only evaluate CT and IM.

For vessel markers, CD34-stained blood tumor vessels were classified into 3 types:

1. Ordinary type vessels: Regularly arranged CD34-positive blood vessels in clusters.
2. Lymphocyte-infiltration type: Disrupted CD34-positive vessels with significant lymphocyte infiltration (lymphoepithelioma-like hepatic carcinoma)^13^
3. VETC: CD34-positive vessels that encapsulate tumor clusters.

For vascular classification, we use the following criteria:

First, vessels are identified as VETC-positive if VETC structures account for more than 5%.

Second, if TILs exceed 10%, the vessels are classified as lymphocyte-infiltrated. Finally, vessels that do not meet either condition are defined as conventional vessels. The cases that reached PCR will not be evaluated.

### Multiple immunofluorescence staining

Multiple immunofluorescence was performed on a series of 5.0 µm histological tumor sections from a representative tumor sample. The sections were treated using an Opal fIHC kit (PerkinElmer). The antibody staining procedure was consistent across all sections. The sections were subjected to back-staining with DAPI (Vector Laboratories). The multiple immunofluorescence plates included CD8, CD34, and CA9. A thinner antibody (Dako, Germany) was used to dilute all antibodies. The secondary antibody was processed using the ImmPRESS HRP (peroxidase) Polymer Cancer Detection Kit (Vector Laboratories). A 1 × plus amplification thinner (PerkinElmer/Akoya Biosciences, 01752) was used to dilute the TSA reagent.

### Statistical methods

Statistical analyses were performed using IBM SPSS Statistics version 26 (IBM Corporation). Laboratory blood test data were categorized as positive or negative, based on clinical values. For markers without established clinical cutoff values, a median-based classification of low or high was applied.

Differences between the investigated factors were assessed using the Kruskal-Wallis test, and the Wilcoxon rank-sum test was employed to evaluate the differences between factors in multiple nodules within the same patients. Cox’s model was used for survival analyses, and the Kaplan-Meier method was applied to visualize the survival curves. In terms of OS, events were defined as deaths caused by HCC after recurrence, and for DFS, any recurrence of HCC was considered an event. Statistical significance was set at a significance threshold of 2-tailed *p*<0.05.

## RESULTS

### Overall observation of 49 patients with HCC used neoadjuvant treatment

In the overall observation, there were a total of 8 patients with multiple nodules and tumor sizes ranging from 0.6 to 16 cm. Microvessel invasion was observed in 12 cases, and an MPR was achieved in 21 cases (MPR, residual tumor <30%). Additionally, 5 cases achieved PCR. In terms of Edmondson staging, there were 19 cases classified as stage 2, 23 cases as stage 3, and 2 cases as stage 4. The stages of the five cases could not be determined by PCR. In all the cases, there was no positive margin (the minimum value was 0.01 cm). The median necrosis rate was 60%; 16 cases were observed with cell degeneration (Table 1).

**TABLE 1:** Preoperative clinicopathological features of HCC in 2 groups with different neoadjuvant or conversion therapy options

Characteristic	Total, n=49	T, n=26	T+AI, n=23	*p* ^a^
Ages, y^b^	57, (34, 74)	59, (34, 74)	56, (30,70)	0.26
Gender, male^c^	45 (86)	22 (85)	20 (87)	>0.99
BMI, abnormal^c^	16 (32)	9 (39)	7 (39)	>0.99
HBV/HCV^c^	—	—	—	0.47
HBV	44 (90)	23 (88)	21 (91)	—
HCV	1 (2)	0	1 (4.3)	—
Not hepatitis B or C	4 (8)	3 (12)	1 (4.3)	—
Cirrhosis^c^	15 (30)	9 (38)	6 (29)	0.75
Alcohol^c^	5 (10)	2 (8.3)	3 (13)	0.67
ALT, abnormal^c^	17 (35)	9 (35)	8 (35)	>0.99
AST, abnormal^c^	19 (39)	10 (38)	9 (39)	>0.99
PLT, abnormal^c^	21 (43)	10 (38)	11 (48)	0.57
CRP, abnormal^c^	22 (45)	8 (31)	14 (61)	**0.047**
WBC, abnormal^c^	36 (73)	20 (77)	16 (70)	0.75
LP, abnormal^c^	40 (82)	20 (77)	20 (87)	0.47
AFP, abnormal^c^	27 (55)	18 (72)	9 (43)	0.072
CEA, abnormal^c^	1 (2)	1 (4.3)	0	>0.99
CA-199, abnormal^c^	5 (10)	3 (13)	2 (10)	>0.99
CA-125, abnormal^c^	9 (18)	5 (22)	4 (19)	>0.99
BCLC stage^c^	—	—	—	>0.99
B	35 (71)	17 (77)	18 (78)	—
C	10 (20)	5 (23)	5 (22)	—
Maximum diameter of tumor bed (cm)^b^	4.6, (0.6, 16)	4.6, (1.3, 12.0)	7.2, (0.6, 16.0)	**0.025**
Maximum diameter of tumor, (cm)^b^	2, (0, 12)	2.4, (0.0, 9.5)	1.5, (0.0, 12.0)	0.30
Residual tumor percentage, %^b^	40 (0, 100)	40 (0, 100)	20 (0, 100)	**0.048**
Edmondson grade^c^	—	—	—	0.12
2	19 (44)	7 (30)	12 (60)	—
3	22 (51)	14 (61)	8 (40)	—
4	2 (5)	2 (8.7)	0	—
Multiple nodules^c^	8 (16)	3 (12)	5 (22)	0.45
MVI^c^	12 (24)	7 (27)	5 (22)	0.75
Margin (cm)^2^	0.2 (0.01, 5)	0.35 (0.01, 5)	0.2 (0.1, 5)	0.87
Necrosis, %^b^	60 (0, 100)	60 (0, 100)	80 (0, 100)	0.34
Cell degeneration^c^	16 (33)	9 (39)	7 (33)	0.76
TNM Stage^c^	—	—	—	0.75
I/II	18 (37)	9 (33)	9 (42)	—
III/IV	31 (63)	17 (67)	14 (61)	—
MPR^c^	21 (43)	8 (31)	13 (57)	0.088
PCR^c^	6 (12)	3 (12)	3 (13)	>0.99

^a^ Kruskal-Wallis rank-sum test; Fisher exact test.

^b^ Median (range).

^c^ n (%).

Abbreviations: AFP, alpha-feto protein; BCLC, Barcelona Clinic Liver Cancer; CA-125, carbohydrate antigen-125; CA-199, carbohydrate antigen-199; CEA, carcinoembryonic antigen; CRP, C-reactive protein; LP, lymphocytes; MPR, major pathologic response (residual tumor tissue <30); MVI, microvascular invasion; PCR, pathologic complete response (negative for tumor cells); PLT, platelet count; WBC, white blood count.

**FIGURE 2:**
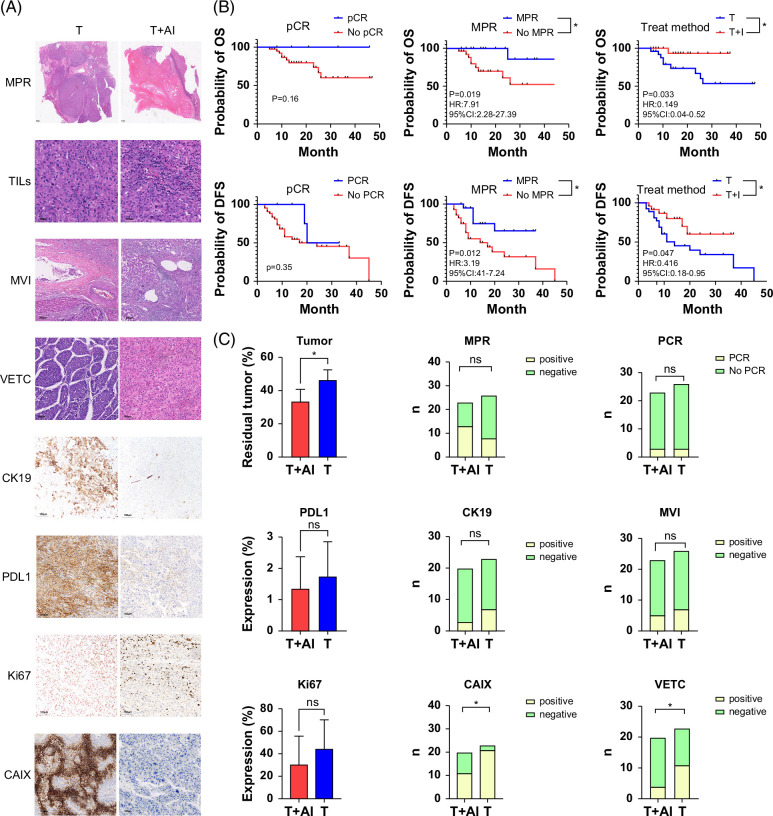
Representative tumor sections and immunohistochemical images of 2 treatment regimens. (A) Representative tumor types and immunohistochemical images for both regimens. (B) KM curves showing the direct relationship between various indicators and prognosis. Top: relationship with OS; bottom: relationship with DFS. (C) Quantitative comparison of tumor indicators between the 2 groups. *p<0.05. Abbreviations: AI, antiangiogenic and immunotherapy; CAIX, carbonic anhydrase 9; DFS, disease-free survival; MPR, major pathologic response; MVI, microvascular invasion; OS, overall survival; PCR, pathologic complete response; VETC, vessels that encapsulate tumor clusters.

In our analysis of residual tumor rates, OS, and DFS in patients, we found that those who achieved MPR after neoadjuvant treatment exhibited a more favorable prognosis than those who did not (*p*=0.019 for OS and *p*=0.012 for DFS). This trend was consistent in patients who underwent PCR, although the difference was not statistically significant (Figure 2, B).

### CD8 TILs are a notable factor with prognosis, T+AI can lead to more curative immune cell infiltration

Cox model was used for survival analyses. All pathological and clinical data were enrolled in the univariate analysis, and *p*<0.05 was accomplished in the multivariate analysis. In terms of OS, CD8+TILs were considered an independent treatment factor (HR=0.13 95%, 95% CI: 0.03–0.58, *p*=0.008), while the presence of satellite nodules was identified as an independent risk factor (HR=4.87, 95% CI: 1.19–20, *p*=0.028) (Table 2). CD8 TILs were also notably associated with DFS. (HR=0.17, 95% CI: 0.04–0.66, *p*=0.01) (Table 3).

**TABLE 2:** Factors associated with overall survival: Univariate and multivariate analysis

	Univariate analysis			Multivariate analysis		
Variables	HR	CI	*p*	HR	CI	*p*
Multiple nodules	5.96	1.57–22.53	0.009	4.87	1.19–20	0.028
Microvascular invasion	4.4	1.26–15.74	0.021	—	—	—
CD8+TILs	0.2	0.05–0.76	0.018	0.13	0.03–0.58	0.008

Abbreviation: TILs, tumor-infiltrating lymphocytes.

**TABLE 3:** Factors associated with recurrence-free survival: Univariate and multivariate analysis

	Univariate analysis			Multivariate analysis		
Variables	HR	CI	*p*	HR	CI	*p*
Satellites nodules	2.92	1.03–8.23	0.043	—	—	—
Necrosis	0.37	0.15–0.91	0.031	—	—	—
Microvascular invasion	3.79	1.63–8.81	0.002	—	—	—
Maximum diameter of tumor	2.58	1.05–6.36	0.039	—	—	—
CD8+TILs	0.17	0.07–0.42	<0.001	0.17	0.04–0.66	0.010
CD8+CT	0.34	0.13–0.88	0.026	—	—	—
MPR	0.28	0.1–0.76	0.013	—	—	—
CD66B+CT	2.94	1.16–7.42	0.023	—	—	—
T staging	2.27	1.21–4.27	0.011	—	—	—
CAIX+	8.25	1.1–61.75	0.040	—	—	—

Abbreviations: CAIX, carbonic anhydrase 9; CT, center of the tumor; MPR, major pathologic response; TILs, tumor-infiltrating lymphocytes.

The cases were categorized into 2 groups (T and T+AI) based on the addition of immunotherapy and antiangiogenic agents. On analyzing the patient prognosis data through survival curve analysis, we identified a distinction in OS/DFS between the 2 patient groups. The T+AI group demonstrated superior OS and DFS compared with the T group. The 2-year survival rate and DFS in the T group were 65.38% and 46.15%, respectively, while those in the T+AI group were 95.65% and 76.26%, respectively, with a notable difference in *p* values (*p*=0.033 for OS and *p*=0.047 for DFS), as illustrated in (Figure 2B). We went through the base of the T and T+AI groups. The CRP level of 10 mg/L in the T+AI group surpassed that in the T group (*p* = 0.047), and the tumor bed was significantly larger in the T+AI group, demonstrating statistical significance between the 2 groups (*p*=0.025). The residual tumor percentage was significantly lower in the T+AI group (*p*=0.048). There were no other differences between the 2 groups in terms of common pathological and clinical data.

From a pathological perspective, we observed the tumor itself and found that, apart from the differences in tumor residual rates between the 2 groups, the positive cases of carbonic anhydrase 9 (CAIX) and VETC in the T group (21/23 and 11/26) were significantly higher than those in the T+AI group (11/20 and 4/20, *p*=0.01 and 0.03, respectively, Figure 2A).

**FIGURE 3:**
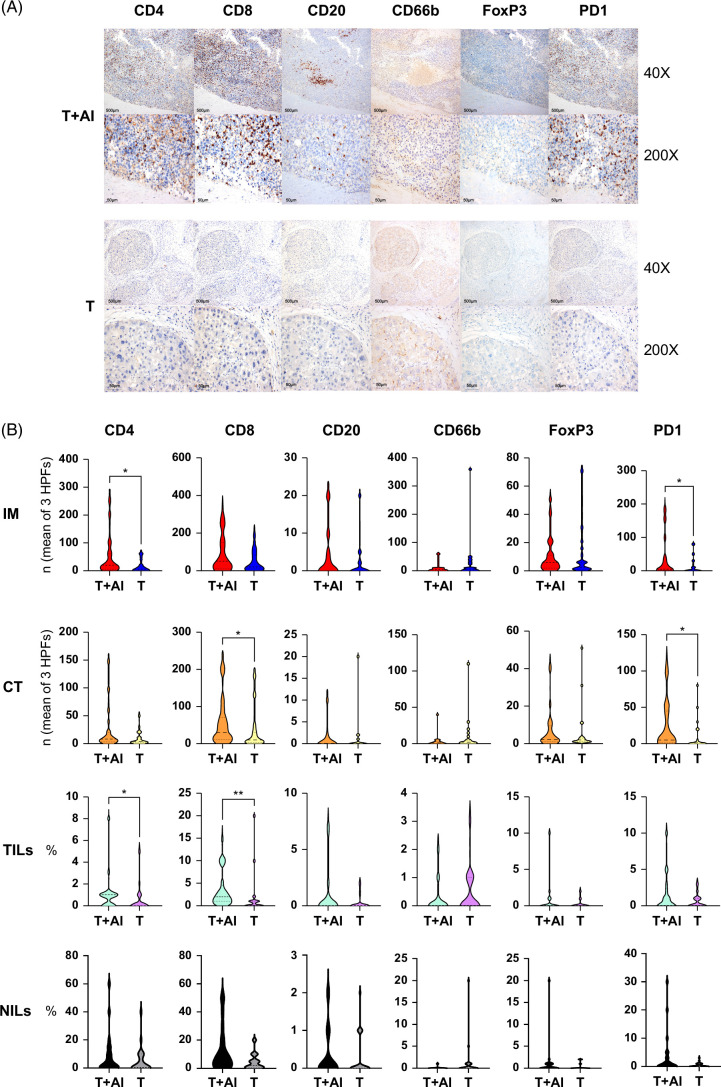
Comparison of representative immune cell staining and assessment methods between 2 treatment regimens. (A) Representative immunohistochemical staining images of both treatment regimens at low magnification (×40, upper) and high magnification (×200, lower). (B) Differences between the 6 indicators under 4 assessment methods between the two treatment regimens. *p<0.05; **p<0.01. Abbreviations: CT, center of tumor; HPF, high-power field; IM, invasive margin; NILs, nontumorous tissue-infiltrating lymphocytes; TILs, tumor-infiltrating lymphocytes percentage.

We found significant lymphocyte infiltration in the T+AI group, especially CD8+T-cell infiltration, whereas almost no lymphocyte infiltration was found in the T group (Figure 3A).

After testing the indicators, we performed an analysis revealing that, in comparison to the T group, the T+AI group exhibited significant enrichment (Figure 3B).

1. CD4+: IM and TILs showed significant enrichment in the group T+AI (*p*=0.014 and 0.032, respectively), while the rest showed no difference.
2. CD8+TILs and CT showed significant enrichment in the group T+AI (*p*=0.014 and 0.008, respectively), whereas the rest showed no difference.
3. PD1+: IM (*p*=0.017) and CT (*p*=0.017) were significantly enriched in the group T+AI.
4. CD20+, CD66b+, FoxP3+: none of them shows a difference.

According to Supplemental Figure S1C, http://links.lww.com/HC9/C71, there appear to be correlations among various indicators. Notably, CD8 expression shows a positive correlation with PD-1, while CD66b exhibits a negative correlation with other immune markers. In addition, PD-L1 Combined Positive Score is positively correlated with PD-1 TILs. Ki-67 demonstrates a negative correlation with CD8 CT and shows certain degrees of association with other markers like PD1 NILs and CD8 NILs.

### The classification of blood vessels can have an impact on the tumor microenvironment, VETC is related to higher CAIX and lower CD8+ TILs

The vascular subtype distribution was as follows: in the T group, there were 8 cases of type 1, 5 cases of type 2, and 11 cases of type 3; in the T+AI group, there were 11 cases of type 1, 5 cases of type 2, and 4 cases of type 3 (Supplemental Figure S1A, http://links.lww.com/HC9/C71).

Kaplan-Meier analysis revealed that VETC (type 3) was significantly associated with worse prognosis, whereas no significant prognostic difference was found between type 1 and type 2 (Supplemental Figure S1B, http://links.lww.com/HC9/C71).

We further conducted a statistical analysis of the 3 types of blood vessels and found that, in addition to morphological differences, the immune microenvironment states were also distinct. We extracted TILs from the 4 indicators for analysis.

**FIGURE 4:**
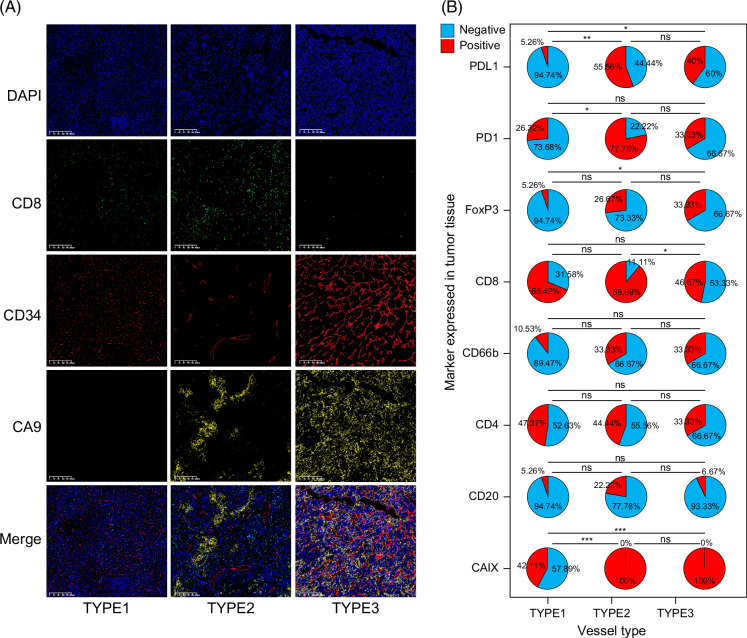
Relationship between vascular types and immune cells. (A) Immunofluorescence showing the relationship between vascular types, tumor phenotypes, and immune cells. (B) Statistical relationship between vascular types and immune cells. *p<0.05; **p<0.01. Abbreviation: CAIX, carbonic anhydrase 9.

HCC with TYPE 1 blood vessels (ordinary type vessels) showed moderate CD8 infiltration (13/19), with low levels of Foxp3 (1/19), PD1 (5/19), and PDL1 (1/19). Notably, CAIX-negative cases were only found in type 1 blood vessel HCC. Type 2 blood vessels (lymphocyte-infiltration type) had the highest positivity rates for CD8 (8/9), FoxP3 (6/9), PDL1 (5/9), and PD1 (7/9), along with the presence of CAIX positivity, and it looks like “dead branch.” Type 3 blood vessel (VETC type) HCC exhibited the lowest CD8 infiltration (7/15), while PDL1(6/15) and CAIX levels were significantly higher than those in type 1 blood vessel HCC (Figure 4A).

Immunofluorescence staining of tumor tissues showed that tumors with TYPE3 blood vessels exhibited significant immune suppression, manifested by lower CD8 levels and significantly higher CA9 staining, whereas, in type 1 and type 2, there was significant lymphocyte infiltration. Type 2 showed obvious lymphocyte infiltration as well as CA9 staining, with a moderate infiltration area (Figure 4B).

### T+AI has a lower rate of CAIX and VETC compared to T, which associated with higher CD8+TILs in tumor

**FIGURE 5:**
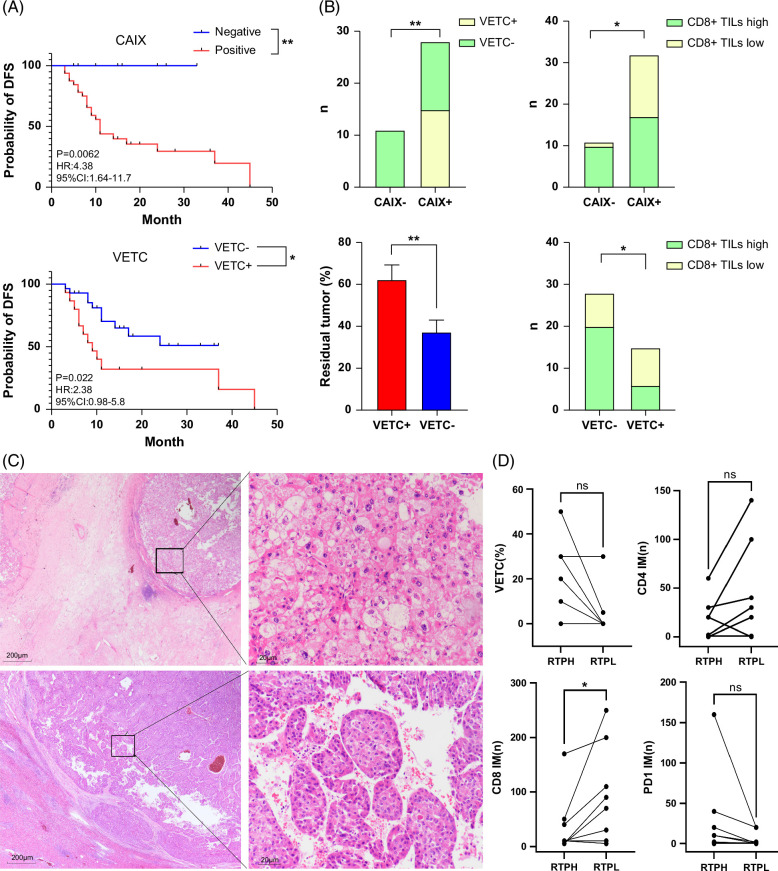
Significance of VETC and CAIX in neoadjuvant therapy. (A) Relationship of CAIX and VETC for DFS. (B) Relationship between VETC, CAIX, and tumors. (C) HE images of multiple nodules in the same patient. Top: nodules without VETC show lower tumor cell residue rates; bottom: tumors with VETC show higher tumor cell residue rates. Left: ×20; right: ×200. (D) Relationship between tumor residue rates and microenvironment indicators in the same patient. *p<0.05; **p<0.01. Abbreviations: CAIX, carbonic anhydrase IX; CT, center of tumor; DFS, disease-free survival; RTPH, residual tumor percentage high; RTPL, residual tumor percentage low; VETC, vessels that encapsulate tumor clusters.

The median DFS of cases positive for CAIX and VETC was significantly lower than that of the negative group (*p*=0.0062 and 0.022). Additionally, we found that CD8+TILs were significantly lower in the CAIX-positive and VETC-positive groups than in the negative group, and CAIX-positive cases had a higher rate of VETC positivity. The proportion of residual tumors in cases that were positive for VETC was also significantly higher than that in the VETC-negative group (Figure 5A).

### VETC affects the efficacy of neoadjuvant therapy as far as the TME of tumor

We conducted an analysis of tumors in 8 patients with multiple nodules and found that even within the same patient, different nodules exhibited markedly inconsistent responses to treatment. Although the difference was not statistically significant, tumors with a lower overall residual rate had fewer VETC, while those with a higher residual rate had more VETC (Figure 5B). Tumors with a higher residual rate also showed significant differences in CD8 IM (Figure 5 C).

## DISCUSSION

TACE is the most commonly used neoadjuvant treatment for HCC.^14^ However, other studies have suggested that the use of TACE alone may not extend the DFS of patients.^15^ Currently, TACE with immunotherapy shows great progress in treatment.^16^ As confirmed in our study, patients treated with the combined use of immunotherapy and TACE showed significantly better prognosis than those treated solely with TACE.

High CD8+TILs levels in specimens have been confirmed to be associated with the prognosis of various cancers, including HCC.^17^,^18^,^19^ Our experiments demonstrated an association between high CD8+TILs levels and the effectiveness of neoadjuvant treatment. Regardless of the use of immunotherapy, cases with high CD8+TILs in tumor specimens had a lower recurrence rate. In our opinion, this is a potential indicator of the effectiveness of neoadjuvant treatment.

The residual rate of tumors serves as a fundamental indicator for assessing the effectiveness of neoadjuvant treatment. In 2 phase II studies, MPR was defined as residual cancer tissue comprising ≤30% of cells.^20^,^21^ Using this definition as the endpoint for neoadjuvant treatment, as mentioned earlier, our observation of H&E-stained sections revealed that cases achieving MPR exhibited significantly better OS and DFS than those who did not. Cases that achieved PCR also demonstrated a notable advantage over non-PCR cases, although due to the limited number of cases, this difference did not reach statistical significance.

Unlike other neoadjuvant therapies, HCC treated with TACE as a neoadjuvant approach inevitably creates a hypoxic environment owing to embolization of the hepatic artery, which affects tumor cell differentiation. The formation of a hypoxic tumor microenvironment has been observed in various solid malignancies, with hypoxia arising from poor vascularization and high metabolic activity. This condition has been linked to the development of chemotherapy resistance, increased tumor aggressiveness, and poor prognosis. Therefore, inhibition of hypoxia is considered a potential method for overcoming drug resistance.

Lai et al^22^ indicated that HCC treated with TACE frequently expresses a “stemness” phenotype, characterized by CA9-positive HCC cells with high co-expression of CK19 and recurrence rate. Furthermore, it has been demonstrated in various cancers,^23^ including HCC,^24^ that CA9 overexpression is closely related to the killing capacity of immune cells and tumor invasiveness, which was also confirmed in our study. In addition, hypoxic and low-pH environments can also have an inhibitory effect on the content of immune cells,^25^ especially CD8+T cells. In our study, the CD8+Tils rate was significantly lower in CAIX-positive cases. Meanwhile, we observed significantly lower CAIX positivity rates in the group treated with antiangiogenic agents. A study suggested that sorafenib downregulates CAIX by upregulating MT1G,^26^ while some studies have suggested that hypoxia itself may impair the sensitivity of HCC to sorafenib.^27^,^28^ Also, a study has reported that sorafenib itself may contribute to the formation of a hypoxic tumor microenvironment.^29^ The hypoxia TME with the relationship between other targeted therapies and hypoxic environments remains largely unexplored. Owing to experimental limitations, further verification is required to elucidate the relationship between these factors.

Moreover, these blood vessels tend to exhibit features characteristic of HCC.^30^ Akiba et al^31^ suggested that the presence of VETC inhibits immune cell infiltration into tumors. In our study, we found that VETC not only correlates with immune cell infiltration but also with post-TACE hypoxia levels and response rates. Furthermore, VETC was significantly less common in the T+AI group. Some studies have indicated that patients with VETC may respond better to sorafenib, although the specific mechanisms are still under investigation. In our observations, we noted that the group receiving targeted therapy combined with TACE had significantly fewer VETC formations than group T.

VETC formation is associated with low levels of immune factors and increased angiogenesis.^31^ We believe that the reduction in VETC following the use of combination-targeted and immune therapy may be related to antiangiogenic factors induced by targeted therapies. In addition, tumors in this group showed more lymphocyte infiltration, which may be related to antigen release triggered by targeted antiangiogenic effects. When we examined the tumor vasculature without VETC, we found a distinctive vascular type that deserves attention. This type showed abundant lymphocyte infiltration and formed a “dead branch” structure, although CA9 positivity persisted in HCC tumors with these vessels. The underlying reason for this remains unclear; it may be related to the state of the response phase when targeted drugs and immunotherapy are used simultaneously.

This study had several limitations. First, the small sample size may introduce errors in tumor microenvironment research. Second, as a retrospective study, we did not obtain pre-neoadjuvant treatment tumor samples, and we could only examine post-treatment tumors to preliminarily assess prognostic markers and their relationship with targeted and immunotherapy. In addition, the inherent heterogeneity of tumors cannot be avoided. Lastly, our analysis was limited to pathological observations without further investigation at the molecular level. Owing to experimental constraints, a deeper exploration of the underlying mechanisms and causes is needed in future studies.

## CONCLUSIONS

In summary, the relationship between HCC and its tumor microenvironment remains a key focus of current research. Our study analyzed the HCC tumor microenvironment under combined targeted and immune therapy from 3 aspects: tumor characteristics, immune microenvironment, and vasculature, and explored their correlation with patient prognosis. First, the significance of CD8 in tumors remains irreplaceable. Furthermore, our investigation revealed a notable correlation between CD8 levels and CAIX expression in tumors, as well as the presence of VETC. This relationship may be one of the factors influencing the efficacy of neoadjuvant therapy and warrants further exploration.

We believe that the improved outcomes of combining targeted therapy with TACE may be attributed to the ability of targeted drugs to mitigate the hypoxic environment induced by TACE, alter vascular morphology, and enhance the efficacy of PD-1 inhibitors, thereby improving patient prognosis.

## Supplementary Material
