# Supplementary material for: Pathological study of the tumor microenvironment after neoadjuvant therapy in hepatocellular carcinoma: Difference of TACE combined with antiangiogenics and immunotherapy
Source: Hepatol Commun. 2025 Aug 29;9(9):e0787. doi: 10.1097/HC9.0000000000000787 (PMC12401379; doi:10.1097/HC9.0000000000000787)
Supplement: Supplementary file 1 [file hc9-9-e0787-s001.docx]

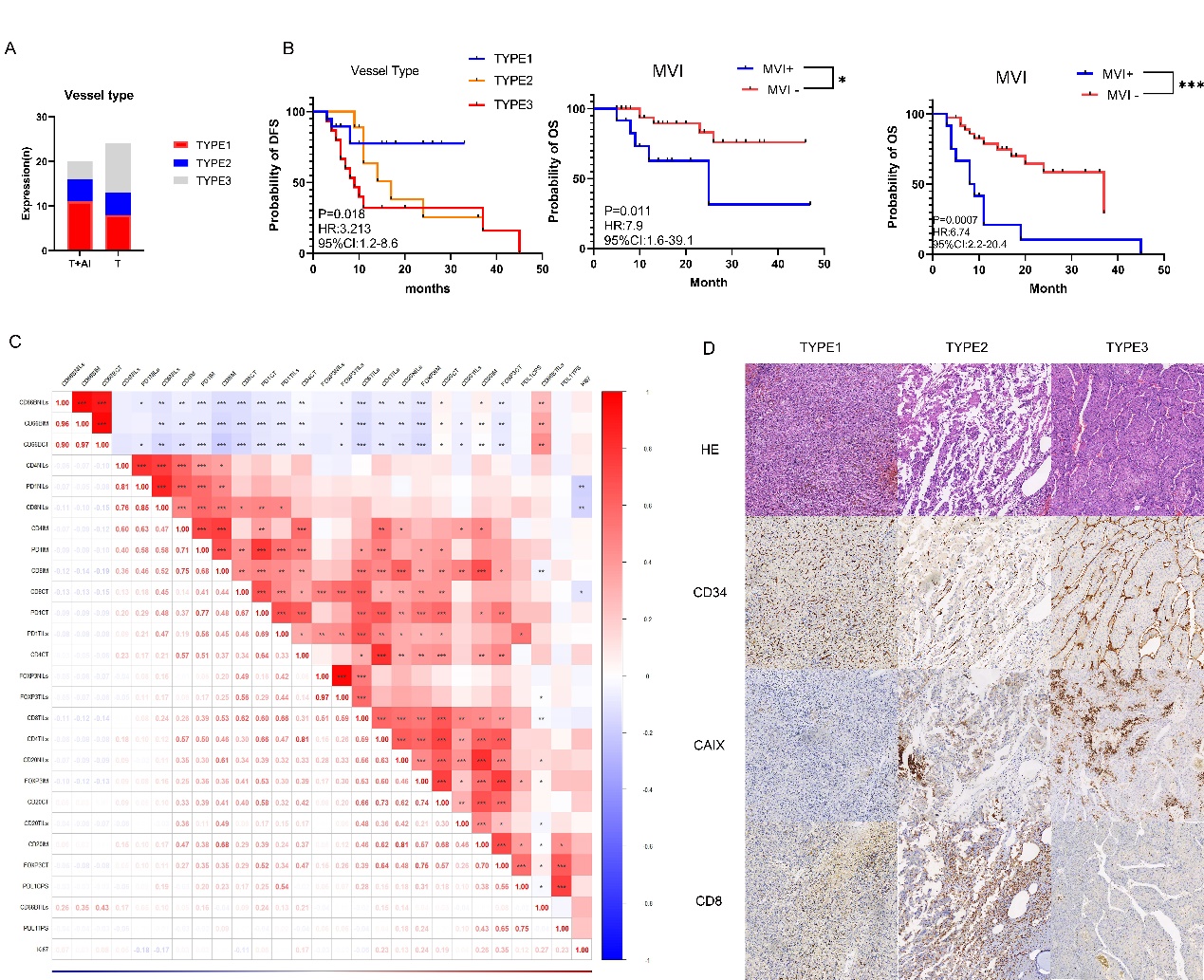


**Figure S1.**

(A) Vessel distribution in two groups;

(B) KM curves showing the direct relationship between various indicators and prognosis.;

(C) Heatmap shows the relationship in various indicators after evaluation. *: p<0.05; **p<0.01; ***P<0.001.

(D) HE and immunohistochemistry showed different type of vessel and marker expression.
